# Prognostic value of the ubiquitin ligase carboxyl terminus of the Hsc70‐interacting protein in postmenopausal breast cancer

**DOI:** 10.1002/cam4.780

**Published:** 2016-06-23

**Authors:** Sasagu Kurozumi, Yuri Yamaguchi, Shin‐ichi Hayashi, Hiromi Hiyoshi, Tetsuji Suda, Tatsuyuki Gohno, Hiroshi Matsumoto, Hiroyuki Takei, Jun Horiguchi, Izumi Takeyoshi, Tetsunari Oyama, Masafumi Kurosumi

**Affiliations:** ^1^ Division of Breast SurgerySaitama Cancer Center780 Komuro, Ina‐machi, Kitaadachi‐gunSaitama362‐0806Japan; ^2^ Research Institute for Clinical OncologySaitama Cancer Center780 Komuro, Ina‐machi, Kitaadachi‐gunSaitama362‐0806Japan; ^3^ Department of Molecular and Functional DynamicsTohoku University2‐1 Seiryo‐chou, Aoba‐kuSendai980‐8575MiyagiJapan; ^4^ Center for Tsukuba Advanced Research AllianceUniversity of Tsukuba1‐1‐1 TennodaiTsukuba305‐8575IbarakiJapan; ^5^ Department of Thoracic and Visceral Organ SurgeryGunma University Graduate School of Medicine3‐39‐22 Showa‐machiMaebashi371‐8511GunmaJapan; ^6^ Department of Diagnostic PathologyGunma University Graduate School of Medicine3‐39‐22 Showa‐machiMaebashi371‐8511GunmaJapan; ^7^ Department of PathologySaitama Cancer Center780 Komuro, Ina‐machi, Kitaadachi‐gunSaitama362‐0806Japan

**Keywords:** Breast cancer, carboxyl terminus of the Hsc70‐interacting protein (CHIP), postmenopausal patients, prognostic factor

## Abstract

The carboxyl terminus of the Hsc70‐interacting protein (CHIP) is considered to induce the ubiquitination and degradation of several oncogenic proteins, and play a role in the inhibition of tumor progression and invasion under experimental conditions. However, the impact of CHIP expression on the prognosis of breast cancer patients has not yet been established. In this study, using an immunohistochemical method, 272 patients with invasive breast cancer were assessed for the expression of CHIP (graded scores 0‐3) and the statuses of biomarkers, such as estrogen receptor (ER), progesterone receptor (PgR), and HER2. The relationships between the statuses of CHIP and biomarkers as well as clinical features were also evaluated, and that between the expression of CHIP and patient prognosis was analyzed. We revealed that the strong expression of CHIP correlated with positive ER (*P *<* *0.001), positive PgR (*P *<* *0.001), and negative HER2 (*P *=* *0.02). In postmenopausal patients, relapse‐free survival (RFS) was significantly better in the high CHIP group than in the low CHIP group (*P *=* *0.042). In addition, RFS and cancer‐specific survival (CSS) were significantly better in patients with ER‐positive/CHIP score 3 tumors than in those with ER‐negative/CHIP score 0 tumors (RFS:* P *=* *0.038, CSS:* P *=* *0.0098). The methylation status of CHIP gene promoter did not always account for the down‐regulation of its expression. In conclusion, the overexpression of CHIP is a potent prognostic factor of a good prognosis in ER‐positive breast cancer patients in the postmenopausal phase.

## Introduction

The carboxyl terminus of the Hsc70‐interacting protein (CHIP) was originally identified as a cochaperone of E3 ligase, which ubiquitinates misfolded or abnormal proteins presented by molecular chaperones such as heat‐shock protein 70 (Hsp70) 1. This protein is considered to be a U‐box‐type ubiquitin ligase that induces the ubiquitination and degradation of its substrates, which include several oncogenic proteins 2, 3. Therefore, CHIP appears to maintain protein homeostasis by controlling chaperone levels during stress and recovery. We previously reported that the expression levels of CHIP mRNA were lower in breast cancer tissue than in normal breast tissue. Furthermore, immunohistochemical staining indicated that the expression levels of CHIP proteins were also lower in breast cancer cells 4. However, the mechanisms underlying the down‐regulated expression of CHIP in breast cancer currently remain unknown. CHIP has been shown to suppress the expression of other oncogenic proteins that enhance anchorage‐independent tumor growth and metastatic potential in breast tumors. Our previous findings indicated that the down‐regulated expression of CHIP led to the accumulation of SRC‐3, thereby resulting in enhanced tumor migration and invasion through increases in Smad and Twist gene transcription 4, 5. Smad and Twist have recently been shown to favor the metastatic dissemination of cancer cells through their abilities to induce epithelial–mesenchymal transition 6, 7. CHIP may also control tumor migration caused by epithelial–mesenchymal transition and suppress the metastatic potential of breast cancer.

Therefore, CHIP controls tumor progression in breast cancer; however, the relationship between CHIP expression and the prognosis of breast cancer patients has not yet been elucidated in detail. In this study, we investigated the relationships between immunohistochemical CHIP expression and several biomarkers as well as that between CHIP expression and the prognosis of patients with invasive breast cancer.

## Patients and Methods

### Patient backgrounds and eligibility

We examined tumor tissue samples from 272 breast cancer patients with invasive carcinoma of no special type, larger than 5 mm, who were diagnosed at Saitama Cancer Center between January 2000 and December 2001. All patients underwent breast‐conserving surgery or modified radical mastectomy without neoadjuvant chemotherapy or neoadjuvant endocrine therapy. Patients with bilateral breast cancer or male breast cancer were excluded. Specimens obtained by surgery were routinely fixed in 20% buffered formalin solution for 3–4 days and embedded in paraffin. Medical records were reviewed for clinicopathological characteristics and follow‐up data for all patients were obtained with a median follow‐up period of 131 months. No human epidermal growth factor receptor 2 (HER2)‐positive patients received adjuvant trastuzumab therapy. We designated patients older than 60 years and/or with no menstruation in the preceding 12 months as postmenopausal.

This study was conducted in accordance with the Declaration of Helsinki, and the protocol of this study was approved by the Institutional Review Board of Saitama Cancer Center. All patients enrolled in this study agreed to the scientific examination of tumor tissues obtained by surgery and provided written comprehensive informed consent.

### Immunohistochemical evaluation of the estrogen receptor (ER), progesterone receptor (PgR), HER2, and Ki67

ER, PgR, HER2 protein, and Ki67 expression levels were examined using immunohistochemistry, and the sources of primary antibodies were as follows: ER (1D5, DAKO, Glostrup, Denmark), PgR (PgR636, DAKO, Glostrup, Denmark), HER2 (HercepTest, DAKO, Glostrup, Denmark), and Ki67 (MIB‐1, DAKO, Glostrup, Denmark). Positive or strong expression was defined by the nuclear labeling index as expression levels of ≥1% for ER, ≥1% for PgR, and ≥30% for Ki67, respectively. On the other hand, HER2 gene amplification was evaluated by a dual in situ hybridization (DISH, Ventana Inc., Tuscon, AZ) method using paraffin‐embedded specimens.

### Immunohistochemical evaluation of CHIP

The CHIP antigen was synthesized in the Department of Life and Environmental Sciences at the University of Tsukuba and the primary antibody against CHIP was produced by Green Space Biomed Co. Tsukuba city in Japan. Immunohistochemical staining was performed on resected tissues. Sections were deparaffinized with xylene and dehydrated through a graded series of ethanol. Sections were immersed in 10 mmol/L citrate buffer (pH 6.0) and boiled for 10 min in a microwave oven to enhance antigenicity. Endogenous peroxidase activity was blocked with 0.3% hydrogen peroxide in methanol. Sections were then incubated for 1 h with a monoclonal antibody against CHIP. An incubation with a secondary antibody (peroxidase‐labeled En Vision polymer reagent; DAKO, Glostrup, Denmark) was performed at room temperature for 30 min. After the visualization of reaction complexes with 0.05% 3,3′‐diaminobenzidine tetrahydrochloride and 0.03% hydrogen peroxide in 50 mmol/L Tris‐HCl buffer (pH 7.6), the sections were examined under a light microscope.

**Figure 1:**
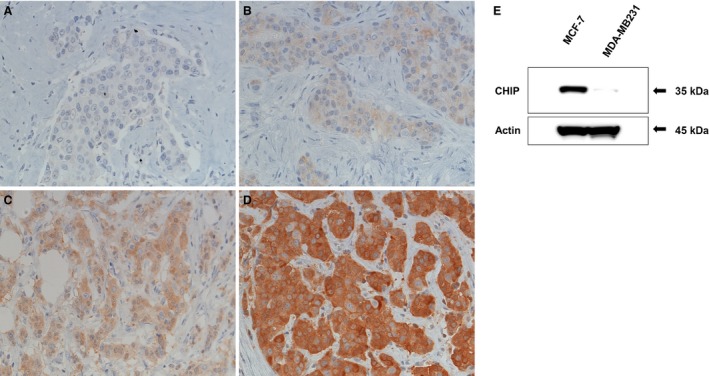
(A‐D) Immunohistochemical findings of CHIP. (A) No staining (score 0), (B) weak staining (score 1), (C) moderate staining (score 2), and (D) strong staining (score 3) for CHIP expression was detected in the cytoplasm of cancer cells. (E) A CHIP protein analysis by western blotting in a breast cancer cell line. The CHIP antibody used in this study clearly detected CHIP proteins in MCF7 cells, but not in MDA‐MB231 cells.

In the immunohistochemical evaluation of CHIP, the staining intensity of the cytoplasm of cancer cells was scored as follows: score 0 (no staining or staining <10% of tumor cells), 1 (weak staining ≥10% of tumor cells), 2 (moderate staining ≥10% of tumor cells), and 3 (strong staining ≥10% of tumor cells), respectively (Fig. 1 a‐d: IHC score 0–3). In addition, tumors with a score of 3 were assigned to the high CHIP group, while tumors with a score of 0, 1, or 2 were placed in the low CHIP group, according to the lowest *P*‐value derived from the survival analysis (Table S1).

### CHIP protein analysis by western blotting

MCF7 and MDA‐MB231 cells were authenticated by the assessment of STR DNA profiling (Promega Corporation, Madison, WI), and were maintained in RPMI1640 medium (Sigma‐Aldrich, St. Louis, MO, USA) containing 10% fetal bovine serum (GIBCO) in 5% CO_2_ at 37°C. Total cell lysates of MCF7 and MDA‐MB231 cells prepared using Lysis‐M (Roche Diagnostics, Indianapolis, IN) were separated by SDS‐PAGE using 10% acrylamide gels (SuperSep^™^, Wako Pure Chemical Industries Osaka, Japan), transferred onto PVDF membranes (Bio‐Rad Laboratories, Inc. Hercules, CA), and the membranes were incubated with an anti‐CHIP or anti‐actin antibody (Sigma‐Aldrich, St. Louis, MO). We previously reported that CHIP protein levels were high in MCF‐7 cells, but not in MDA‐MB231 cells [4] and the same anti‐CHIP antibody was used for the immunohistochemical evaluation performed in this study. We confirmed that this antibody clearly detected CHIP proteins in MCF7 cells, but not in MDA‐MB231 cells (Fig. 1E).

### Methylation analysis by the COBRA assay

In order to elucidate the mechanism responsible for the down‐regulated expression of CHIP, we analyzed the DNA methylation of the promoter of the CHIP gene using the COBRA assay 8. DNA methylation is frequently observed in the gene promoter region with cancer progression in order to silence genes. Several 40‐μm thick sections containing a large tumor site were sliced from formalin‐fixed paraffin‐embedded tissues, and DNA was extracted by the Recover All Total Nucleic Acid Isolation Kit for FFPE (Life Technologies, Carlsbad, CA) according to the manufacturer's instructions. DNA from breast cancer cell lines was extracted using the QIAamp DNA Mini Kit (Qiagen, Mississauga, Ontario, Canada). Extracted DNA was treated with bisulfite using the EpiTect Bisulfite Kit (Qiagen, Mississauga, Ontario, Canada) according to the manufacturer's instructions. Nested PCR was then carried out at 95°C for 2 min, followed by 42 cycles (40 cycles for 2nd PCR) at 95°C for 30 sec, 48°C for 30 sec (1 min for 2nd PCR), 72°C for 1 min, and a final extension of 7 min at 72 °C. The oligonucleotides used in nested PCR were as follows: 1st PCR forward primer 5′‐TTG GAT TTA TTA GGG AGG TT‐3′, 2nd PCR forward primer 5′‐TTA AGT TGT TAG GTT AGT AG‐3′, and common reverse primer 5′‐CTA AAC TAC CAT TCT AAA AC‐3′. This region contained CpG sites. The PCR amplicon was electrophoresed on 2% agarose gels and DNA was extracted from the correct‐sized band using the QIAquick Gel Extraction Kit (Qiagen, Mississauga, Ontario, Canada). The extracted PCR products were subsequently treated with the restriction enzyme Cla I (Takara, Kusatsu, Japan) for 2 hr at 37°C, and reelectrophoresed on polyacrylamide gels. The gels were stained by SYBR green, and the separated (methylated, 140 bp and 108 bp) and unseparated (unmethylated, 248 bp) bands were detected using the Image Analyzer LAS4000 system (Fuji Film Co., Ltd., Tokyo, Japan).

### Clinical outcome investigation

We investigated the relationships between CHIP expression, clinicopathological features, and the statuses of biomarkers, such as ER, PgR, and HER2. Relapse‐free survival (RFS) and cancer‐specific survival (CSS) were analyzed between patient groups divided by the degree of CHIP expression. In addition, a multivariate survival analysis including CHIP expression, the menopausal status, clinical T factor (cT), clinical node status (cN), histological grade (HG), ER status, PgR status, and HER2 status was performed. We also evaluated the relationship between CHIP and Ki67.

### Statistical analysis

Statistical analyses were conducted using Stat Mate 4 for Windows (ATMS, Tokyo, Japan) and SPSS v15.0 (SPSS Inc., Chicago, IL). The chi‐squared and Fisher's exact tests were used to analyze the relationships between the expression of CHIP and clinicopathological characteristics. The Kaplan–Meier method and log‐rank test were used to estimate RFS and CSS functions. RFS was defined as the time length from the date of surgery to the date of any recurrence (including ipsilateral breast recurrence). CSS was determined as the time length from the day of surgery to the time of death caused by breast cancer. The Cox proportional hazards regression model and associated 95% confidence interval (CI) were assessed for several clinicopathological factors including CHIP. *P*‐values <0.05 were defined as significant.

## Results

### Patient characteristics

The median age of the 272 patients enrolled in this study was 54 years (age range: 25–87 years), with 117 (43.0%) patients in the premenopausal state and 155 (57.0%) in the postmenopausal state. Sixty‐nine patients (25.4%) had T1 tumors (AJCC Staging System); 166 (61.0%) T2; 21 (7.7%) T3; and 16 (5.9%) T4. The clinical lymph node status of 143 (52.6%) patients was N0, while 111 (40.8%), 17 (6.3%), and 1 (0.4%) patients had N1, N2, and N3 lymph node statuses, respectively. Forty‐six (16.9%) patients had stage I breast cancer, while 118 (43.4%), 71 (26.1%), 21 (7.7%), 15 (5.5%), and 1 (0.4%) patients had IIA, IIB, IIIA, IIIB, and IIIC stage cancers, respectively. A total of 148 (54.4%) patients received adjuvant chemotherapy, while 164 (60.3%) received adjuvant endocrine therapy (Table S2).

### Immunohistochemical expression of the CHIP protein

As shown in Figure 1A‐D, the immunohistochemical staining of CHIP was observed in the cytoplasm of cancer cells, while no or weak staining was observed in normal mammary gland cells. The distribution of patients stratified by CHIP expression scores was as follows: score 0, 49 patients (18.0%); score 1, 91 (33.5%); score 2, 83 (30.5%); and score 3, 49 (18.0%). The number of patients in the low CHIP expression (scores 0, 1, and 2) group was 223 (82.0%) and in the high CHIP expression group (score 3) was 49 (18.0%) (Table 1).

**Table 1:** Relationship between CHIP expression, biomarkers, and histological grade

CHIP	Score 0	Score 1	Score 2	Score 3	*P*	All (*n* = 272)
Histological grade						
Grade 3	36	51	40	27	0.044	154
Grade 1/2	13	40	43	22		118
ER						
Negative (<1%)	34	25	15	7	<0.001 (−2.07E‐10)	81
Positive (≥1%)	15	66	68	42		191
PgR						
Negative (<1%)	34	32	23	16	<0.001 (0.00016)	105
Positive (≥1%)	15	59	60	33		167
HER2						
Negative	34	78	72	44	0.022	228
Positive	15	13	11	5		44

ER, estrogen receptor; PgR, progesterone receptor; HER2, human epidermal growth factor receptor 2.

### Relationship between CHIP expression and ER and PgR statuses

The distribution of patients according to the CHIP score and ER‐positive rate was as follows: score 0, 15 out of 49 patients (30.6%); score 1, 66 out of 91 (72.5%); score 2, 68 out of 83 (81.9%); and score 3, 42 out of 49 (85.7%). CHIP protein expression levels were significantly higher in patients with ER‐positive tumors (*P *<* *0.001; Table 1). The ER‐positive rate of the low CHIP group was 149 out of 223 patients (66.8%) and that of the high CHIP group was 42 out of 49 (85.7%). The distribution of patients according to the CHIP score and PgR‐positive rate was as follows: score 0, 15 out of 49 patients (30.6%); score 1, 59 out of 91 (64.8%); score 2, 60 out of 83 (72.3%); and score 3, 33 out of 49 (67.3%), respectively. CHIP protein expression levels were significantly higher in patients with PgR‐positive tumors (*P *<* *0.001; Table 1). The PgR‐positive rate of the low CHIP group was 134 out of 223 patients (60.1%) and that of the high CHIP group was 33 out of 49 patients (67.3%).

### Relationship between CHIP expression and HER2 and HG statuses

Based on the CHIP score and HER2‐positive rate, the distribution of patients was as follows: score 0, 15 out of 49 patients (30.6%); score 1, 13 out of 91 (14.3%); score 2, 11 out of 83 (13.3%); and score 3, 5 out of 49 (10.2%), respectively. CHIP protein expression levels were significantly higher in patients with tumors negative for HER2 (*P *=* *0.022; Table 1). Based on the CHIP score and HG3, the distribution of patients was as follows: score 0, 36 out of 49 patients (73.5%); score 1, 51 out of 91 (56.0%); score 2, 40 out of 83 (48.2%); and score 3, 27 out of 49 (55.1%), respectively (*P *=* *0.044; Table 1).

### Prognostic significance of CHIP protein expression

**Figure 2:**
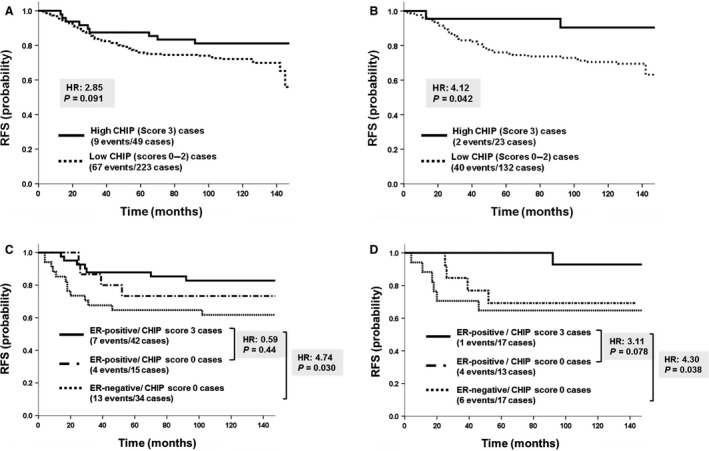
Relapse‐free survival (RFS) curve stratified by CHIP expression. (A) The survival of the high CHIP (score 3) group was slightly better than that of the low CHIP (scores 0, 1, or 2) group (*P *=* *0.091). (B) In postmenopausal patients, RFS was significantly better in the high CHIP group than in the low CHIP group (*P *<* *0.05). (C) RFS was significantly better in the estrogen receptor(ER)‐positive/CHIP‐score 3 group than in the ER‐negative/CHIP‐score 0 group (*P *<* *0.05). (D) In postmenopausal patients, RFS was significantly better in the ER‐positive/CHIP‐score 3 group than in the ER‐negative/CHIP‐score 0 group (*P *<* *0.05).

The median RFS was 130 months (range: 1–151 months), and the median follow‐up duration was 131 months (range: 4–154 months). RFS tend to be better in the high CHIP group than in the low CHIP group (HR = 2.85, *P *=* *0.091; Fig. 2A), whereas CSS was not (HR = 0.45, *P *=* *0.50). Among premenopausal patients, no significant difference was observed in survival between the high CHIP and low CHIP groups (RFS: HR = 0.17, *P *=* *0.68; CSS: HR = 0.33, *P *=* *0.56). However, among postmenopausal patients, RFS was significantly better in the high CHIP group than in the low CHIP group (RFS: HR = 4.12, *P *=* *0.042; CSS: HR = 3.01, *P *=* *0.083; Fig. 2B). In postmenopausal ER‐positive patients, RFS tend to be better in the high CHIP group than in the low CHIP group (RFS: HR = 3.69, *P *=* *0.055; CSS: HR = 3.03, *P *=* *0.082); however, in postmenopausal ER‐negative patients, no significant differences were observed in RFS or CSS between the high CHIP and low CHIP groups (RFS: HR = 0.33, *P *=* *0.57; CSS: HR = 0.27, *P *=* *0.61).

RFS was significantly better in the ER‐positive/CHIP score 3 group than in the ER‐negative/CHIP score 0 group (HR = 4.74, *P *=* *0.030; Fig. 2C), whereas CSS tend not to (HR = 3.70, *P *=* *0.054). Furthermore, among premenopausal patients, no significant difference was observed in survival rates between the ER‐positive/CHIP score 3 group and ER‐negative/CHIP score 0 group (RFS: HR = 1.54, *P *=* *0.21; CSS: HR = 0.30, *P *=* *0.58); however, in postmenopausal patients, survival rates were significantly better in the ER‐positive/CHIP score 3 group than in the ER‐negative/CHIP score 0 group (RFS: HR = 4.30, *P *=* *0.038; CSS: HR = 6.68, *P *=* *0.0098; Fig. 2D).

### Relationship between prognosis and clinicopathological characteristics of tumors in postmenopausal breast cancer

A univariate analysis (Table 2) suggested that the low expression of CHIP, negative ER status (positive vs. negative; RFS: HR = 1.24, *P *=* *0.50; CSS: HR = 1.96, *P *=* *0.075), high histological grade (grade 1/2 vs. 3; RFS: HR = 1.80, *P *=* *0.074; CSS: HR = 2.12, *P *=* *0.073), positive HER2 status (positive vs. negative; RFS: HR = 1.87, *P *=* *0.085; CSS: HR = 2.25, *P *=* *0.052), high clinical T factor (clinical T 1/2 vs. clinical T 3/4; RFS: HR = 2.51, *P *=* *0.015; CSS: HR = 3.54, *P *=* *0.0026), and positive clinical node status (negative vs. positive, RFS: HR = 5.32, *P *<* *0.0001; CSS: HR = 3.82, *P = *0.0014) may be worse prognostic factors in this study. On the other hand, a multivariate analysis (Table 2) showed that CHIP expression was an independent prognostic marker for RFS (RFS: HR = 4.94, *P *=* *0.030; CSS: HR = 6.55, *P *=* *0.068). Based on the results of the multivariate analysis, the HER2 status was identified as an independent prognostic marker for RFS (RFS: HR = 4.03, *P *=* *0.014; CSS: HR = 2.77, *P *=* *0.085). The clinical lymph node status was also identified as an independent prognostic marker for RFS and CSS in the multivariate analysis (RFS: HR = 5.63, *P *<* *0.0001; CSS: HR = 3.63, *P *=* *0.0039).

**Table 2:** Result of univariate and multivariate survival analysis of clinicopathologic variable influences including CHIP expression in postmenopausal breast cancer

Characteristics	RFS				CSS			
	Univariate		Multivariate		Univariate		Multivariate	
	HR (95% CI)	*P*	HR (95% CI)	*P*	HR (95% CI)	*P*	HR (95% CI)	*P*
CHIP								
Low vs. high	3.91 (0.94–16.17)	0.060	4.94 (1.17–20.95)	0.030	4.91 (0.67–36.12)	0.12	6.55 (0.87–49.18)	0.068
Ki67								
≥30% vs. <30%	1.12 (0.59–2.11)	0.73	1.14 (0.49–2.62)	0.77	1.24 (0.58–2.64)	0.59	0.74 (0.28–1.94)	0.55
ER								
Negative vs. positive	1.24 (0.66–2.33)	0.50	0.49 (0.17–1.41)	0.19	1.96 (0.93–4.12)	0.075	1.10 (0.34–3.51)	0.88
HG								
Grade 3 vs. Grade 1–2	1.80 (0.95–3.41)	0.074	1.46 (0.69–3.09)	0.33	2.12 (0.93–4.80)	0.073	1.47 (0.56–3.85)	0.43
HER2								
Positive vs. negative	1.87 (0.92–3.80)	0.085	4.03 (1.32–12.27)	0.014	2.25 (0.99–5.12)	0.052	2.77 (0.87–8.82)	0.085
Clinical tumor size								
T3‐4 vs. T1‐2	2.51 (1.19–5.26)	0.015	1.10 (0.48–2.52)	0.82	3.54 (1.56–8.05)	0.0026	1.91 (0.77–4.75)	0.16
Clinical nodal status								
N1‐3 vs. N0	5.32 (2.61–10.84)	<0.0001	5.63 (2.64–12.00)	<0.0001	3.82 (1.68–8.67)	0.0014	3.63 (1.51–8.69)	0.0039

RFS, relapse free survival; CSS, cancer‐specific survival (CSS); HR, hazard ratio; 95% Cl, 95% confidence interval; ER, estrogen receptor; HG, histological grade; HER2, human epidermal growth factor receptor 2.

### Prognostic significance of CHIP protein expression in luminal B‐like breast cancer

**Figure 3:**
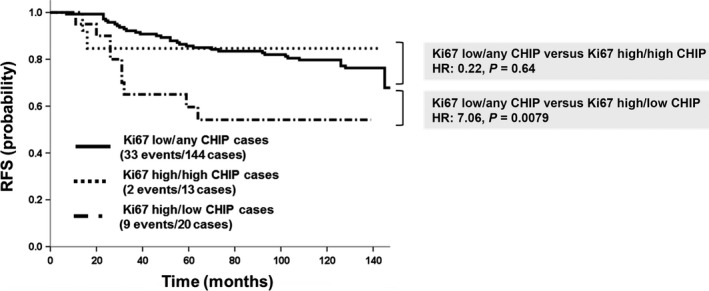
Survival curves of low Ki67/any CHIP, high Ki67/low CHIP, and high Ki67/high CHIP groups in ER‐positive/HER2‐negative breast cancer patients. RFS was significantly worse in the high Ki67/low CHIP group than in the low Ki67/any CHIP group (*P *<* *0.01). On the other hand, no significant differences were noted in survival rates between the high Ki67/high CHIP group and low Ki67/any CHIP group (*P *=* *0.64).

The intrinsic subtypes of breast cancer were determined by the immunohistochemical expression of ER, PgR, and HER2. ER‐positive and HER2‐negative patients were further categorized into luminal A (low Ki67) and B (high Ki67) subtypes by the Ki67 labeling index, which is considered to be a significant prognostic marker. In ER‐positive/HER2‐negative patients, RFS and CSS were worse in the luminal B/low CHIP group than in the luminal A/any CHIP group (HR = 7.06, *P *=* *0.0079; CSS: HR = 7.32, *P *=* *0.0068; Fig. 3). On the other hand, no significant differences were observed in survival rates between the luminal B/high CHIP group and luminal A/any CHIP group (RDS: HR = 0.22, *P *=* *0.64; CSS: HR = 0.092, *P *=* *0.76; Fig. 3).

### Methylation of the CHIP promoter in breast cancer

**Figure 4:**
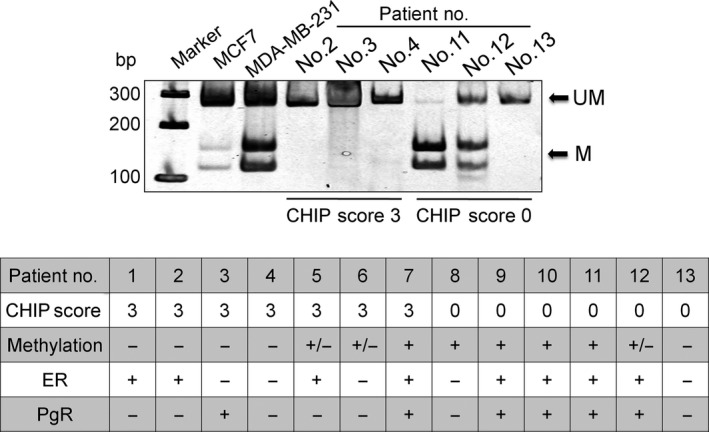
CHIP score and methylation status of the CHIP gene promoter in breast cancer. Methylation of the CHIP promoter was analyzed by COBRA for breast cancers with CHIP scores 0 and 3. UM = unmethylation; M = methylation. Patients with partial methylation were shown as +/−. ER and PgR statuses for each breast cancer are also shown.

A correlation was observed between CHIP expression and the methylation status in only eight out of the 13 samples (61.5%) analyzed (Fig. 4), suggesting that other mechanisms such as histone deacetylation may also be involved in the regulation of CHIP expression.

## Discussion

In this study, we clarified that the strong expression of CHIP correlated with positive ER, positive PgR, and negative HER2, and the overexpression of CHIP may be a prognostic factor of a good prognosis in ER‐positive breast cancer patients. In addition, we identified the expression of CHIP as a potent prognostic factor for RFS in postmenopausal patients with ER‐positive invasive breast cancer. In the analysis of CSS, the lowest *P*‐value was 0.07 when the cut‐off value was set at a score of 0 versus 1–3. This result suggests the possibility of a poor prognosis for CHIP‐negative (score 0) patients.

A correlation was found between patients strongly expressing CHIP and ER‐positive and/or PgR‐positive breast cancer. ER is known to play important roles in the development and progression of breast cancer. It controls the expression of various genes and proteins through genomic and nongenomic pathways. In the genomic pathway, estrogen signals are mediated through ER. ER also functions as a transcription factor to target genes including PgR 9, 10. ER was previously shown to be maintained in a ligand‐binding conformation by Hsp70‐based chaperones 11. In the absence of CHIP, ER may be sequestered within a stable chaperone protein complex consisting of Hsp70. CHIP has been suggested to play a role in regulating the stability and turnover of ER and is closely associated with ER genomic activities 12, 13.

On the other hand, we also revealed an inverse correlation between CHIP protein and HER2 protein levels. HER2 is a member of the ErbB‐protein family and acts in a signaling network in the cellular membrane 14. HER2‐containing dimers have been shown to enhance downstream signaling 14, 15. The mechanism underlying the down‐regulated expression of HER2 signaling involves the removal of receptors from the cell surface in the course of initial ligand‐induced endocytosis and subsequent sorting to the lysosome for degradation through ubiquitination 16. Previous studies suggested that CHIP bridged the interaction between HER2 and Hsp70 as identified with the degradation of activated HER2 9. CHIP may play pivotal roles in controlling HER2 protein levels on the cell surface. It is speculated that the overexpression of CHIP in cancer cells may accelerate the process of endocytosis and degradation of HER2 protein by ubiquitination.

Growth factor receptors on the cell membrane related to signal crosstalk with the activation of ER, such as the epidermal growth factor receptor and insulin growth factor‐1 receptor 9, 17, are also mainly mediated by the ubiquitin–proteasome pathway. One potential mechanism involves CHIP targeting Hsp70‐interacting proteins for ubiquitination and proteasomal degradation. Recent studies reported that CHIP attenuated transmembrane receptor‐mediated gene transcription 2, 18, 19, 20. Therefore, CHIP may contribute to the regulation of functional growth factor receptor levels in ER‐positive breast cancer.

Differences in behaviors in breast cancer development between premenopausal and postmenopausal patients have been reported, and some investigators suggested the difference of grade of tumor‐induced angiogenesis 21, 22, 23, 24 and indicated the difference of incidence of lymph node metastasis. In addition, it is well known that plasma estrogen levels of premenopausal women are significantly higher than that of postmenopausal women. Therefore, it is speculated that inhibitory effect of CHIP to breast cancer growth might be actualized more strongly in postmenopausal state because of condition of low plasma estrogen level 25.

Wang et al. reported that the down‐regulation of CHIP in the late stages of colorectal cancer was mainly caused by the methylation of its promoter 26. In this study, CHIP expression levels were not always associated with methylation of the CHIP promoter, suggesting that it is also regulated through mechanisms other than methylation such as histone deacetylation. Further studies are needed in order to ascertain the mechanisms underlying the impaired expression of CHIP in breast cancer cells.

The expression of CHIP was identified as a potent prognostic factor in luminal B‐like breast cancer in this study. Perou et al. initially proposed a molecular classification for breast cancer. Using a cDNA microarray of 38 breast cancer cases, this group defined a list of intrinsic genes 27, 28. Moreover, a larger cohort of breast cancer patients revealed that the luminal subgroup may be divided into luminal A and B 29. Luminal B was previously shown to have a more aggressive phenotype, higher HG, and more proliferative index such as Ki67 than luminal A 30, 31, 32. The prognosis of patients with luminal B tumors was also found to be worse than those with luminal A tumors despite treatments with hormonal therapy 33. These discrepancies between luminal A and B may be due to different estrogen‐related intracellular signaling pathways in breast cancer cells. However, the mechanisms responsible for luminal B breast cancer, particularly survival, proliferation, and metastasis, have not yet been elucidated in detail. Therefore, the molecular therapeutic target of luminal B is currently under investigation 34, and the up‐regulation of CHIP may improve the survival of patients with luminal B breast cancer. We reported that the novel agent, 2‐(4‐hydroxy‐3‐methoxyphenyl)‐benzothiazole (YL‐109) induced CHIP transcription and inhibited breast cancer cell growth and invasiveness in vitro 35. Further biological research regarding the ability of this new agent to up‐regulate the expression of CHIP is warranted, and CHIP is expected to become one of the therapeutic targets in luminal B‐like breast cancer.

## Conflict of Interest

None declared.

## Supporting information


**Table S1.** Survival analysis according to the degree of CHIP expression.
**Table S2.** Patient and tumor characteristics at baseline.
