# Supplementary material for: Prognostic value of the ubiquitin ligase carboxyl terminus of the Hsc70‐interacting protein in postmenopausal breast cancer
Source: Cancer Med. 2016 Jun 23;5(8):1873–82. doi: 10.1002/cam4.780 (PMC4971916; doi:10.1002/cam4.780)
Supplement: Supplementary file 1 — Table S1. Survival analysis according to the degree of CHIP expression. Table S2. Patient and tumor characteristics at baseline. [file CAM4-5-1873-s001.docx]

| **Table S1. Survival analysis according to the degree of CHIP expression** | | | | |
| --- | --- | --- | --- | --- |
|  | **RFS** | | **CSS** | |
| **CHIP Score Cut-off point** | **HR** | ***P*-value** | **HR** | ***P*-value** |
| 0 vs 1-3 | 1.91 | 0.17 | 3.23 | 0.07 |
| 0-1 vs 2-3 | 0.01 | 0.92 | 0.14 | 0.71 |
| 0-2 vs 3 | 2.85 | 0.091 | 0.45 | 0.50 |
| Abbreviations: RFS= Recurrence-free survival; CSS= Cancer-specific survival; HR= Hazard Ratio. | | | | |

| **Table S2. Patient and tumor characteristics at baseline.** | | | |
| --- | --- | --- | --- |
|  | | **(n= 272)** | |
| **Menopausal status at diagnosis** | | |  |
| Premenopausal | | 43.0 (117) | |
| Postmenopausal | | 57.0 (155) | |
| **Clinical tumor size** |  |  |  |
| T1 | | 25.4 (69) | |
| T2 | | 61.0 (166) | |
| T3 | | 7.7 (21) | |
| T4 | | 5.9 (16) | |
| **Clinical nodal status** |  |  |  |
| N0 | | 52.6 (143) | |
| N1 | | 40.8 (111) | |
| N2 | | 6.3 (17) | |
| N3 | | 0.4 (1) | |
| **Clinical stage** |  |  |  |
| I | | 16.9 (46) | |
| IIA | | 43.4 (118) | |
| IIB | | 26.1 (71) | |
| IIIA | | 7.7 (21) | |
| IIIB | | 5.5 (15) | |
| IIIC | | 0.4 (1) | |
| **Type of surgery** |  |  |  |
| Breast-conserving surgery | | 79.4 (216) | |
| Mastectomy | | 20.6 (56) | |
| **Axillary management** |  |  |  |
| Sentinel lymph node biopsy alone | | 47.8 (130) | |
| Axillary lymph node dissection | | 51.1 (139) | |
| No surgery | | 1.1 (3) | |
| **Adjuvant Chemotherapy** | |  |  |
| Yes | | 54.4 (148) | |
| No | | 45.6 (124) | |
| **Adjuvant Endocrine therapy** | |  |  |
| Yes | | 60.3 (164) | |
| No | | 39.7 (108) | |
